# Single Cell Gene Co-Expression Network Reveals FECH/CROT Signature as a Prognostic Marker

**DOI:** 10.3390/cells8070698

**Published:** 2019-07-10

**Authors:** Xin Chen, Lingling Hu, Yuan Wang, Weijun Sun, Chao Yang

**Affiliations:** 1 Guangdong Key Laboratory of IoT Information Technology, School of Automation, Guangdong University of Technology, Guangzhou 510006, China; 2 Faculty of Health Sciences, University of Macau, Macau 999078, China

**Keywords:** FECH, CROT, co-expression, single cell, prostate cancer, prognostic

## Abstract

Aberrant activation of signaling pathways is frequently observed and reported to be associated with the progression and poor prognosis of prostate cancer (PCa). We aimed to identify key biological processes regulated by androgen receptor (AR) using gene co-expression network from single cell resolution. The bimodal index was used to evaluate whether two subpopulations exist among the single cells. Gene expression among single cells revealed averaging pitfalls and bimodality pattern. Weighted gene co-expression network analysis (WGCNA) was used to identify modules of highly correlated genes. Twenty-nine gene modules were identified and AR-regulated modules were screened by significantly overlapping reported androgen induced differentially expressed genes. The biological function “generation of precursor metabolites and energy” was significantly enriched by AR-regulated modules with bimodality, presenting differential androgen response among subpopulations. Integrating with public ChIP-seq data, two genes FECH, and CROT has AR binding sites. Public in vitro studies also show that androgen regulates FECH and CROT. After receiving androgen deprivation therapy, patients lowly express FECH and CROT. Further survival analysis indicates that FECH/CROT signature can predict PCa recurrence. We reveal the heterogeneous function of “generation of precursor metabolites and energy” upon androgen stimulation from the perspective of single cells. Inhibitors targeting this biological process will facilitate to prevent prostate cancer progression.

## 1. Introduction

The new cases of prostate cancer (PCa) are estimated up to ~164,700 at the United States in 2018, as the most prevalent cancer type and the second leading cause of cancer death among the males [1]. Androgen receptor (AR) is essential in the growth and development of both normal and cancer prostate gland. Androgen deprivation is the standard of care for men with PCa [2]. Unfortunately, recurrence is emerged in a considerable proportion of patients despite the level of castrated androgen. It has been reported that increased AR activity drives therapeutic resistance in advanced prostate cancer [3]. Therefore, it is crucial to dissect the mechanism of AR regulation network.

Bulk population based RNA-seq may mask the presence of intratumoral heterogeneity, which may consequently hide important biological insights [4]. Single cell RNA-seq offers opportunities to renew the understanding of diseases and its biological processes at an unprecedented resolution. Subclonal heterogeneity has been unravelled in triple negative breast cancer through single-cell RNA-seq. One of the subpopulation is characterized by activation of glycosphingolipid metabolism and innate immunity pathways, which leads to poor outcomes [5]. Single cell analysis has facilitated to inspire novel and deeper insight into the expression of marker genes [6]. Gli3 is reported as a negative regulator of a subpopulation of taste cells [7]. COX7B is also identified as a novel platinum resistance gene based on single-cell RNA-seq analysis [8].

Network medicine, an extension of network biology, aims to develop a global understanding of the key components in the network generated based on phenotype-specific biomedical data. It has been extensively and gainfully applied to identify phenotype-specific biomarkers [9]. The gene co-expression network (GCN) covers human genome, ultimately keeping all the genes detected by sequencing. GCN facilitates the successful identification of an AR variant-driven gene module based on the meta-analysis of microarray datasets of PCa [10]. Besides, the GCN was used for function prediction of biomarker candidates [11]. Each gene expression profile generated from RNA sequencing is comprised of an expression of ten thousands of genes in the detected samples. The GCN constructed for all the detected genes is too large to interpret due to its high sparsity and dimension. It is the impetus to identify gene modules from the complex network by decomposing the GCN. Weighted correlation network analysis (WGCNA) identifies gene clusters using hierarchical clustering and can extract meaningful biological information for the underlying phenotype [12]. In the weighted GCN of WGCNA, the correlation weight was raised by power value to keep the high correlation and simultaneously to penalize the low correlation. WGCNA has been successfully applied to single cell transcriptome data to identify cell subpopulation and subtype- or phenotype-specific markers [13,14,15]. Besides gene or lncRNA expression patterns, repeatome exhibits dynamic pattern by applying WGCNA to single cells in early human embryonic development [16].

A recent study presents that LNCaP cells respond heterogeneously to androgen-deprivation therapy. The resistant subpopulation to androgen deprivation therapy has been found and is characterized by enhanced cell cycle activity [17]. Therefore, in this study, we aimed to find out the key biological processes regulated by AR and its corresponding androgen regulated genes from the single cell perspective. WGCNA was used to identify modules of highly correlated genes. We focus on the modules significantly involved in AR regulation. The AR regulated genes in the target module were further validated in public ChIP-seq dataset. Survival analysis further determined the prognostic potential of candidate genes.

## 2. Materials and Methods

### 2.1. Data Preprocessing

We downloaded the processed expression (RPKM) profiles of LNCaP cells generated by single cell RNA-seq from GEO (accession ID: GSE99795). The dataset contains 144 LNCaP cells from 0 h untreated, 12 h untreated and 12 h R1881 treated conditions. There are 48 cells under each condition. We filtered out genes that lowly expressed (RPKM < 1) in more than two thirds of all the samples. The expression profiling contains expression value of 10 445 genes in 144 LNCaP cells. Then, the gene co-expression network (GCN) was constructed based on this gene expression profile.

The raw data of RNA-seq datasets were downloaded from NCBI SRA to cross validate the expression of FECH and CROT. All the raw data were processed into expression values (FPKM) as previously described [18]. The survival analysis for candidate markers was performed based on the GSE70769 and MSKCC dataset.

### 2.2. Weighted Correlation Network Analysis (WGCNA)

WGCNA (version 1.49) was performed as we described previously [12,19]. Gene co-expression network was constructed based on the symmetric matrix generated by the pairwise correlation of gene expression. For gene *i* and *j*, their expression similarity is defined as $S_{ij}=\left|cor(x_{i},x_{j})\right|$. *x_i_* and *x_j_* represents the expression profile of gene *i* and *j*, respectively. In the weighted correlation network, adjacency $a_{ij}$ is defined as:

$$a_{ij}=s_{ij}^{\beta },$$

where *β* ≥ 1. To obtain the adjacency matrix, the *β* was chosen from 1 to 20. Considering the scale-free topology characteristic of the network (R^2^ = 0.9), the power *β* = 4 was selected (Figure S1). The adjacency matrix was then converted into a topological overlap matrix (TOM). In constructing the clustering tree, 1-TOM is used as a distance measure for hierarchical clustering. In the clustering tree, branch cutting algorithm “Dynamic Tree Cut” is used to define gene modules, in which the genes have highly similar expression pattern.

### 2.3. Identifying Genes with AR Binding Sites Based on ChIP-seq Dataset

ChIP-seq data was downloaded from GEO database and bowtie2 was used to mapping the raw data to hg19 reference with default parameter. Samtools was used to convert the .sam to .bam with -q 20 to filter out reads with low quality. MACS2 pileup with --extsize 147 and ucsc-bedgraphtobigwig were performed to pileup the data and transform to .bw files for further visualization.

### 2.4. Androgen Regulated Genes

The algorithm SCDE [20] was used to identify differentially expressed genes (DEGs) between 12-h androgen-treated and 12-h untreated cells [17]. 272 DEGs [17] were regarded as androgen regulated genes (ARGs). To further determine the gene modules regulated by androgen, the hypergeometric test was used to test if the 272 ARGs significantly overlapped with the genes in the module.

$$p=\sum_{x\geq n}\frac{C_{N}^{x}C_{M-N}^{m-x}}{C_{M}^{m}},$$

where *M* was the number of genes for WGCNA; N was the number of ARGs (*N* = 272); m was the number of genes in one gene module; n was the number of module genes in the 272 ARGs. If the *p*-value was smaller than 0.05, the gene module was regarded as regulated by androgen.

### 2.5. Gene Ontology Annotation and Enrichment Analysis

DAVID [21] v6.8, an online web tool, was utilized for functional enrichment analysis along three aspects of Gene Ontology: biological process (BP), molecular function (MF) and cellular component (CC). EASE Score, a modified Fisher Exact *p*-value, was used to evaluate whether the genes in the module significantly overlapped with genes involving in a specific biological function. The smaller the *p*-value is, the more enriched by the interested genes are. The enrichment threshold of *p*-value was set to 0.05.

### 2.6. Statistic Method for Differential Expression and Survival Analysis

The expression difference of candidate gene between two groups is compared by Manne-Whitney U test, one type of nonparametric tests. Kaplan-Meier and Cox regression analyses are utilized to assess the prognostic significance of mRNAs. The statistical analysis was performed using R. For the survival analysis of gene signature, the Z-score for each gene was calculated. The mean Z-score of the gene set is regarded as the expression of the gene signature.

### 2.7. Gene Expression Bimodality Analysis

Bimodality expression was performed using the R package, SIBER [22]. First, a normal mixture model (‘NL’) was specified on the log2 transformed RPKM expression values to fit the gene expression distribution into a two component mixture model (component 1 and 2). Next the average values (mu1 and mu2) were calculated. Other parameters were also obtained including variance values (sigma1, sigma2) and corresponding proportion of the component 1 and 2 (pi1 and pi2). Then the bimodal index (BI) was used to assess the potential bimodality of individual gene based on the equation:

$$BI=\frac{\sqrt{pi_{1}\cdot pi_{2}}\cdot \left|mu_{1}-mu_{2}\right|}{\sqrt{pi_{2}\cdot sigma_{1}^{2}+pi_{1}\cdot sigma_{2}^{2}}}.$$

## 3. Results

### 3.1. Bimodal Gene Expression Among Single Cells Reveals Averaging Pitfalls

**Figure 1:**
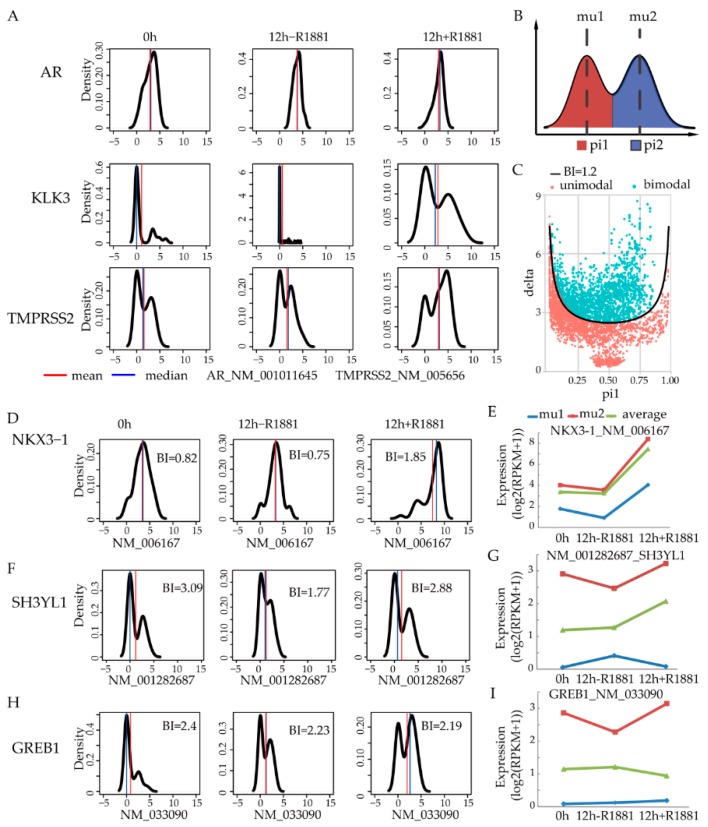
Bimodal gene expression among single cells reveals averaging pitfalls. (**A**) Expression distribution of marker genes (AR, KLK3, and TMPRSS2) in single cells; (**B**) The bimodality index (BI) is used to characterize the possibility of two subgroups among the single cells. Pi1 and pi2 represent the ratio of the lower group and higher group, respectively. Mu1 and mu2 denote the average level of individual groups; (**C**) Genes with BI > 1.2 (blue dots) are regarded as bimodally expressed genes; (**D**) Expression of known androgen regulated genes and corresponding BI value under each condition for NKX3-1; (**E**) Subpopulation expression of NKX3-1; (**F**) Expression trend of SH3YL1; (**G**) Subpopulation expression of SH3YL1; (**H**) Expression trend of GREB1; (**I**) Subpopulation expression of GREB1. Line charts show the average expression level in all single-cell (green line), higher group (red line), and lower group (blue line).

We looked into the expression of AR and known ARGs such as KLK3 and TMPRSS2. It was shown that AR expression did not change (Figure 1A). In contrast, the expression of KLK3 increased significantly upon androgen stimulation. Moreover, there are obvious two expression peaks with distinct expression level after treated by R1881. One group of cells does not express KLK3, while the other group of cells highly expresses KLK3. For another known ARG, TMPRSS2 always presented two expression peaks under all three conditions. After androgen stimulation, more cells increased the expression of TMPRSS2, while fewer cells didn’t express TMPRSS2. This indicates that not all the cells synchronously increase the expression of ARGs, highlighting the cell heterogeneity in response to androgen stimulation. Therefore, we tried to use bimodal expression pattern to characterize the cell heterogeneity. The algorithm SIBER [22] fitted the gene expression distribution into two log-normal distributions (component 1 and 2). For both components, the average values (mu1 and mu2) and corresponding proportions (pi1 and pi2) of these two subpopulations were also calculated (Figure 1B). Finally, a value called bimodal index (BI) was used to determine the extent of bimodal expression. The larger the BI value is, the more likely the cell heterogeneity is. We calculated the BI for all genes across all three conditions. As recommended by Tong P [22], the genes with a BI > 1.2 were regarded as bimodal (Figure 1C).

One of the major issues of a bimodal gene is that its averaged expression value may not truly represent its two subpopulations. To address this, we compared the averaged expression level of bimodal ARGs. As shown in Figure 1D, NKX3-1 showed a bimodal pattern upon androgen stimulation. When we extracted the component 1 and 2 of NKX3-1, the expression change was similar among average expression level of mu1, mu2, and global average expression (Figure 1E). SH3YL1, a recently reported ARG [23], increased its expression at 12 h when compared with 0 h (Figure 1F). Intriguingly, mu1 for SH3YL1 decreased at 12 h with androgen stimulation. However, due to the averaging with mu2, the mean expression level of all cells increased (Figure 1G). Because of the average effect, decreased expression of mu2 at 12h without androgen stimulation was also masked in the average level. For GREB1, a known ARG, it is consistently bimodally expressed at all three conditions (Figure 1H). Mu2 increased after R1881 treatment (Figure 1I). However, GREB1 expression slightly decreased from the average expression of all the cells. The opposite expression change of mu1 or mu2 for known ARGs shows conflicting trend of average effect from traditional bulk cells.

### 3.2. Twenty-Nine Gene Modules Are Identified Using WGCNA

**Figure 2:**
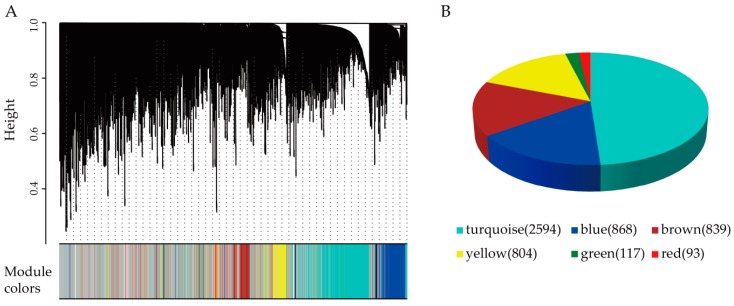
Gene modules were detected by the weighted correlation network analysis (WGCNA). (**A**) The genes are divided into modules and named by colors. (**B**) Gene number in the top six modules are shown in the bracket.

WGCNA was used to construct the gene co-expression network (GCN) based on the dataset GSE99795. Genes with expression level larger than 1 (RPKM > 1) in more than one third of samples were considered for further analysis. The gene expression profiles, comprising of 10,445 genes in 144 samples, were log2 transformed and subjected to WGCNA. As shown in Supplementary Figure S1, power 4 was chosen as the soft threshold to identify co-expressed gene modules (for details, see the section of Materials and Methods). The clustering dendrogram of genes was shown in Figure 2A. Twenty-nine gene modules were identified. As the "grey" module was reserved for unassigned genes, we thus focused on modules except the grey module. The top six modules containing the most number of genes were color-coded as turquoise, blue, brown, yellow, green, and red (Figure 2B).

### 3.3. Modules Significantly Regulated by Androgen Receptor

Androgen receptor (AR) plays important roles in tumorigenesis of prostate cancer. Aaron Horning [17] et al. identified 272 androgen induced differentially expressed genes (DEGs) by comparing the gene expression levels with or without androgen (R1881) stimulation, using SCDE package to identify differential genes based on single-cell RNA-seq data. We would like to use these 272 DEGs as androgen regulated genes (ARGs) to determine which modules are most likely to be regulated by AR. 

**Figure 3:**
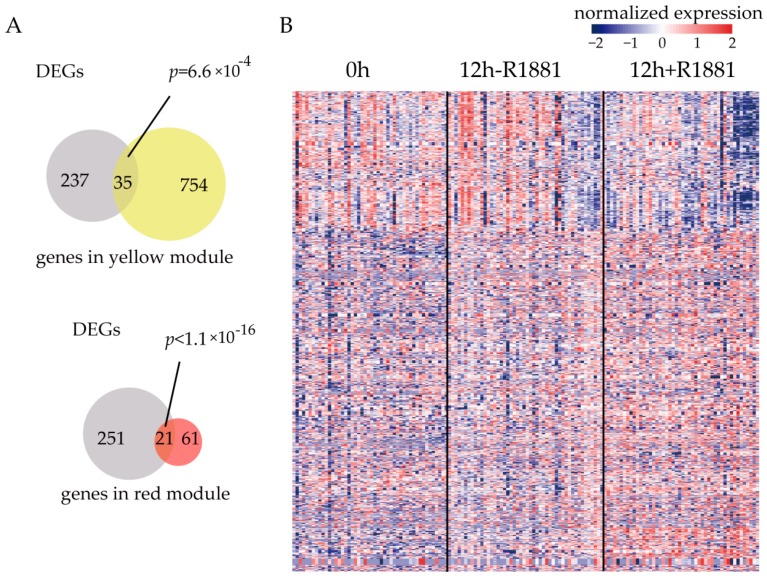
Gene modules significantly regulated by androgen receptor. (**A**) The yellow and red modules significantly overlapped with differentially expressed genes (DEGs) between 12 h + R1881 vs 12 h − R1881 from GSE99795. (**B**) The expression heatmap of genes in the yellow and red module.

For each module, we calculated the number of ARGs. The hypergeometric test was used to determine whether the ARGs significantly enriched in the module. From the *p-*values, we found that, the yellow and red modules significantly overlapped with ARGs (*p* = 6.6 × 10^−4^ for the yellow module and *p* < 1.1 × 10^−16^ for the red module, Figure 3A). For genes in these two modules, the expression heat map was shown in Figure 3B. There are more up-regulated ARGs in cancer than those down-regulated ones.

### 3.4. Biological Functions of Modules Significantly Regulated by Androgen Receptor

**Figure 4:**
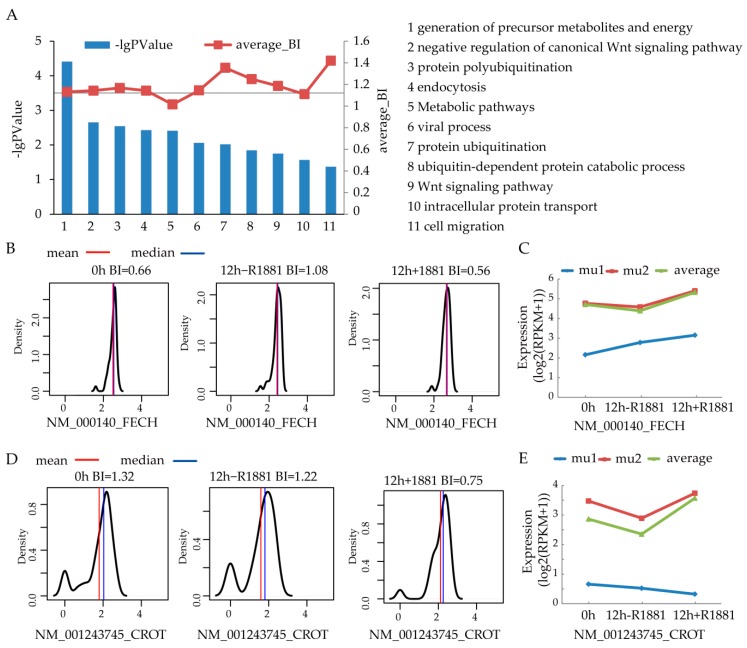
Interested functional terms and genes. (**A**) The significantly enriched terms with high BI value. The bar represents the minus log10 *p-*value of enrichment significance. The text on the right is the names of terms corresponding to x-axis in the bar chart; (**B**) Expression trend of FECH; (**C**) Subpopulation expression of FECH; (**D**) Expression trend of CROT; (**E**) Subpopulation expression of CROT.

The genes in the yellow and red modules significantly overlapped with ARGs from single cell RNA-seq dataset [17]. We next did functional enrichment analysis to explore the functions of genes in these two gene modules, based on Gene Ontology and KEGG pathway. The significant functional terms (*p* < 0.05) with more than 10 genes in red and yellow modules were selected. The significance of function enrichment is shown in Figure 4A. Because the bimodal gene expression commonly existed among single cells (Figure 1), we aimed to find the function terms that showed most bimodally expressed pattern. The average_BI value (Figure 4A) for a function term is the average BI of module genes in this term. Based on the enrichment significance and average_BI value of each term, we used the R package “RobustRankAggreg” to rank the terms. We used a different method to prioritize the term list, the term “generation of precursor metabolites and energy” ranked as the top function (Supplementary Table S1).

There are 11 genes from the yellow module that participate the term “generation of precursor metabolites and energy”. Jiang et al. developed the androgen responsive gene database (ARGDB) to provide integrated knowledge on androgen-controlled genes [24]. Among these 11 genes, FECH and CROT are novel androgen regulated genes according to ARGDB. As the members of bimodally expressed terms, we then looked at the expression pattern of FECH and CROT in the single cells. As shown in Figure 4B, FECH is a unimodal gene, with BI value smaller than 1.2. However, the expression trend of two subpopulations is different. When comparing the 0 h untreated and 12 h untreated condition, FECH is up-regulated in the one subpopulation (mu1) while it is slightly down-regulated in the other subpopulation (mu2) (Figure 4C). In contrast to FECH, CROT is a bimodal gene under two conditions (Figure 4D), with a BI value larger than 1.2. The expression of CROT after androgen stimulation is different for two subpopulations (Figure 4E).

### 3.5. FECH and CROT Expression in Patients

**Figure 5:**
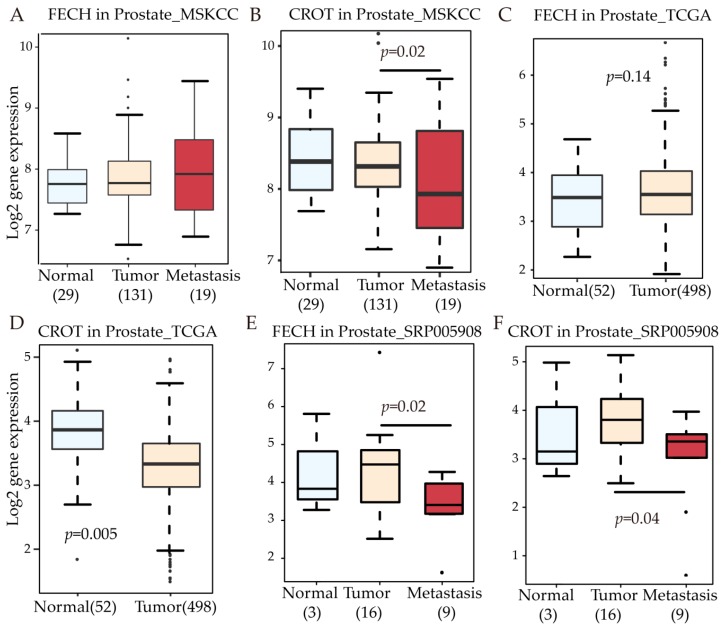
FECH and CROT expression in prostate cancer. (**A**) FECH and (**B**) CROT expression in prostate dataset MSKCC; (**C**) FECH and (**D**) CROT expression in prostate dataset TCGA; (**E**) FECH and (**F**) CROT expression in prostate dataset SRP005908.

FECH and CROT participated in the bimodal function “generation of precursor metabolites and energy” from our single cell analysis. We would like to know how both genes expressed in patients of prostate cancer. The expression level of FECH (Figure 5A) and CROT (Figure 5B) is not significantly changed between normal and primary tumor based on the MSKCC dataset. Similarly, FECH does not significantly change between normal and primary tumor based on the TCGA dataset (Figure 5C). But in the TCGA dataset, CROT is significantly differentially expressed in tumor samples (Figure 5D, *p* = 0.005). In the dataset SRP005908, both FECH (Figure 5E) and CROT (Figure 5F) decreased their expression in metastatic patients compared with primary samples.

### 3.6. FECH/CROT Signature Is Potent Prognostic Marker Regulated by AR

**Figure 6:**
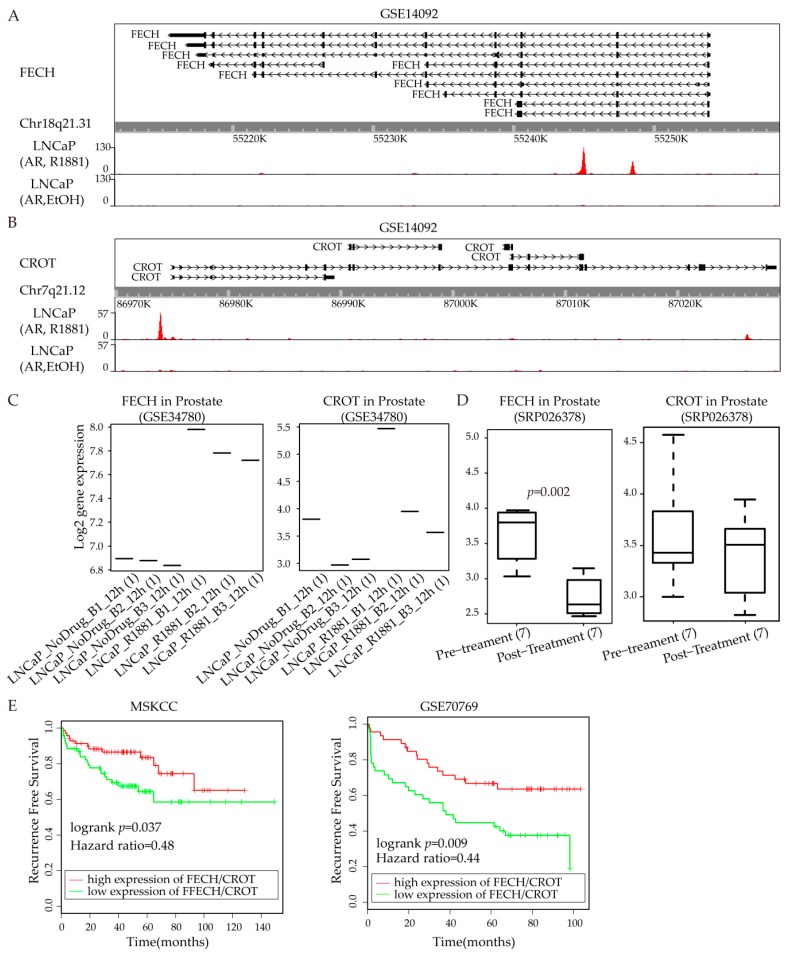
FECH and CROT are potent prognostic markers regulated by AR. (**A**) FECH has AR binding sites (ARBS) from public ChIP-seq dataset (GSE14092); (**B**) CROT has ARBS from public ChIP-seq dataset (GSE14092); (**C**) Expression of FECH and CROT before and after R1881 stimulation in LNCaP cells (GSE34780); (**D**) FECH and CROT expression after androgen ablation therapy (AAT) in prostate cancer patients (SPR026378); (**E**) FECH/CROT signature is a potent prognostic marker of prostate cancer based on survival analysis of the MSKCC dataset (left) and GSE70769 (right).

As shown in Figure 3A, FECH and CROT are androgen regulated genes according to expression profile. We further explored whether FECH and CROT had AR binding sites. Based on the public dataset from AR ChIP-seq (GSE14092), we found that FECH and CROT both had strong and dense AR binding sites (ARBS) (Figure 6A, Figure 6B). We also validate FECH and CROT as ARGs via public in vitro cell experiments. According to the androgen stimulation experiment in the public dataset (GSE34780), FECH is up-regulated after androgen stimulation (Figure 6C, the left panel). The expression of CROT generally increases after R1881 treatment (Figure 6C, the right panel). These results indicate that FECH and CROT are candidate androgen regulated genes. Furthermore, we would like to explore the expression of FECH and CROT after androgen ablation therapy (AAT). The expression of both genes decreased after AAT as expected (Figure 6D). Moreover, we further explore the prognostic potential of FECH and CROT. As shown in Figure 6E, the FECH/CROT signature can predict biochemical recurrence of prostate cancer patients in the MSKCC dataset (Figure 6E, left panel) and GSE70769 (Figure 6E, right panel). See details for survival analysis in the Materials and Methods section.

## 4. Discussion

A previous study [17] identified ten cell cycle related genes which can stratify the cell subpopulation likely to develop aggressive progression leading to the recurrence of prostate cancer (PCa). WGCNA decomposed the GCN into 29 small components (gene modules). Modules regulated by androgen receptor were defined by significantly overlapping gene members with those androgen induced DEGs from a recent publication [17]. The biological functions of these modules are implied by functional enrichment analysis based on Gene Ontology (GO) and KEGG pathway. The bimodal expression pattern of genes has been revealed by the expression profile from the single cell resolution. Considering both the enrichment significance (*p*-value) and bimodal pattern of functional terms, RobustRankAggreg package was used to prioritize target terms. We found out the AR regulated metabolic process “generation of precursor metabolites and energy” showed heterogeneous response to hormone stimulation.

The high bimodality of “generation of precursor metabolites and energy” indicates the switching metabolic attributes during androgen response. Treating the T-lymphoblastic leukemia cells by daunorubicin, doxorubicin, and mitoxantrone, the differentially expressed proteins involved in the generation of precursor metabolites and energy [25]. In yeast, the sodium selenite exposure resulted in the dysregulation of genes belonging to the function "generation of precursor metabolites and energy" [26]. FECH and CROT, two androgen regulated genes, are also involved in this function. The results also showed FECH (Figure 6A) and CROT (Figure 6B) was in the yellow module with AR binding sites. As the terminal enzyme in the heme biosynthesis pathway, MEK inhibition suppressed FECH activity to convert Protoporphyrin IX into heme in various human normal and cancer cell lines [27]. Among the function terms shown in Figure 4A, FECH also participates in the metabolic pathways. Several known androgen regulated genes are also included in this function. For example, UAP1 is up-regulated in the early stages of prostate cancer. UAP1 could protect prostate cancer cells against inhibitors of N-linked glycosylation [28]. It has also been reported that glycosylation is an androgen-regulated process essential for the viability of prostate cancer cells [29]. More recently, Jennifer Munkley has summarized that the glycosylation is potentially a global target for androgen regulation in prostate cancer cells [30]. DHCR24, also known as seladin-1, is regarded as androgen induced genes due to its consistent over-expression in prostate cancer than normal prostate tissue [31]. The overexpression of AR attenuated the methylseleninic acid inhibition of ARGs including DHCR24. Therefore, as a component in the same pathway, FECH may also play important roles in androgen regulation in prostate cancer. This is consistent with the results that FECH has ARBS (Figure 6A) and its expression increases after androgen stimulation (Figure 6C). For the patients received AAT, FECH expression decreases as expected (Figure 6D). After applying WGCNA on the single cell RNA-seq dataset generated from androgen stimulus experiment, we identified FECH as a novel ARG involving in the function of “generation of precursor metabolites and energy”. As shown in Figure 5, FECH does not significantly differentially expressed in primary prostate cancer compared with normal prostate. Therefore, there is low possibility to identify FECH as an ARG from differential analysis of the expression profile from prostate patients.

CROT is reported to involve in fatty acid β-oxidation [32]. Similar to FECH, CROT does not consistently differentially express between the primary prostate tumor and normal prostate (Figure 5). Copy number gains were acquired in the genes all located on chromosome 7 including CROT, which potentially drives the development of docetaxel resistance in breast cancer cells [33]. Moreover, CROT is dysregulated in the p53-dependent Molt-4 leukemia cells after γ-irradiation [34]. Among nine of the 11 multidrug-resistant ovarian cancer cell line variants, the drug-resistant gene MDR1 (ABCB1) is co-expressed with multiple genes located in 7q21, including CROT and CDK6 [35]. These indicate that CROT may play important roles in drug-resistance in multiple cancer cell lines. Kaplan–Meier survival analysis show that FECH is a biochemical recurrence (BCR) marker in dataset GSE70769 (Figure S2A), but not in the dataset MSKCC (Figure S2B). In contrast, CROT is a BCR marker in dataset MSKCC but not in GSE70769 (Figure S2C,D). The combination signature of FECH/CROT can predict BCR in both datasets (Figure 6E). 

In the dataset GSE99795 [17], from the average single cell level, FECH was slightly down-regulated after 12 h untreated (fold change = 0.93). As shown in the line plot of Figure 4C, there are two subpopulations among the single cells. One group (mu2) represented the subpopulation in which the average expression level was significantly higher than that of the global average levels of all the cells. The other group (mu1) represented the lower expressed cells. After 12 h untreated, the expression of FECH in group A (red line in Figure 4C) slightly decreased while in group B its expression increased (blue line in Figure 4C, fold = 1.29). Therefore, we can find out the low expressed subpopulation from single cell level that, which has been masked from traditional bulk studies.

In summary, we discovered that the biological process “the generation of precursor metabolites and energy” biomodally functioned upon response to androgen stimulation. As members in this function, FECH and CROT are both regulated by AR. Moreover, in vitro experiments also validate the regulation relationship between AR and FECH/CROT. The FECH/CROT signature is a potent BCR predictor of PCa. Inhibitors which can target FECH or CROT may facilitate to prevent PCa progression.

## Supplementary Materials

The following are available online at https://www.mdpi.com/2073-4409/8/7/698/s1, Figure S1: Parameters used to determine the threshold (power value), Figure S2: Survival analysis for FECH and CROT, Table S1: Rank functional terms using R package RobustRankAggreg.
