# Supplementary material for: Single Cell Gene Co-Expression Network Reveals FECH/CROT Signature as a Prognostic Marker
Source: Cells. 2019 Jul 10;8(7):698. doi: 10.3390/cells8070698 (PMC6678878; doi:10.3390/cells8070698)
Supplement: Supplementary file 1 [file cells-08-00698-s001.zip › cells-538361 supplementary xml/Figure S1.pdf]

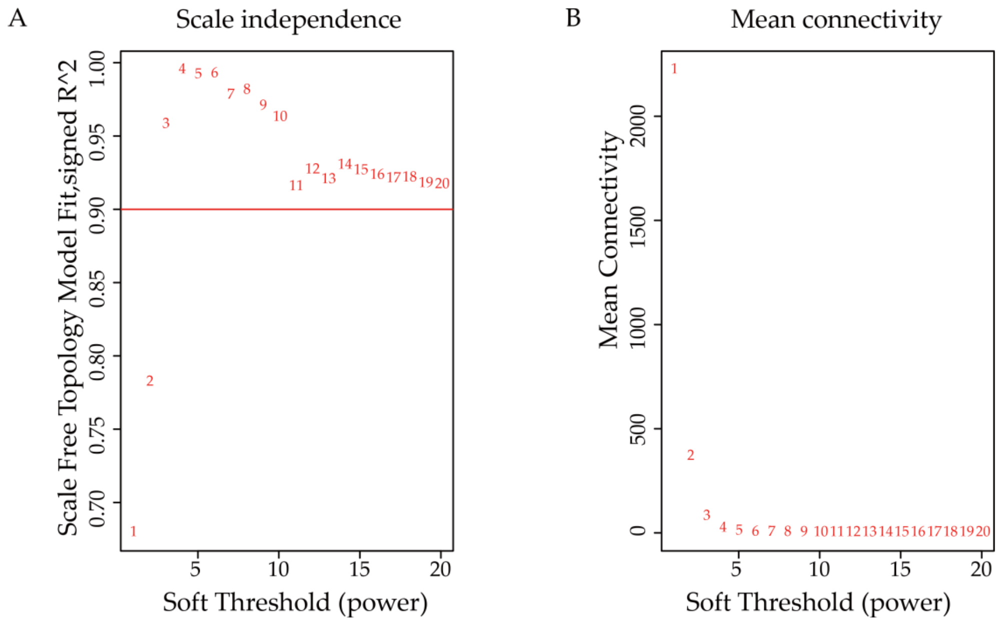

Figure S1. Parameters used to determine the threshold (power value). (**A**) Scale-free topology index and (**B**) mean connectivity were used to determine the soft threshold.
