# Supplementary material for: Single Cell Gene Co-Expression Network Reveals FECH/CROT Signature as a Prognostic Marker
Source: Cells. 2019 Jul 10;8(7):698. doi: 10.3390/cells8070698 (PMC6678878; doi:10.3390/cells8070698)
Supplement: Supplementary file 1 [file cells-08-00698-s001.zip › cells-538361 supplementary xml/Figure S2.pdf]

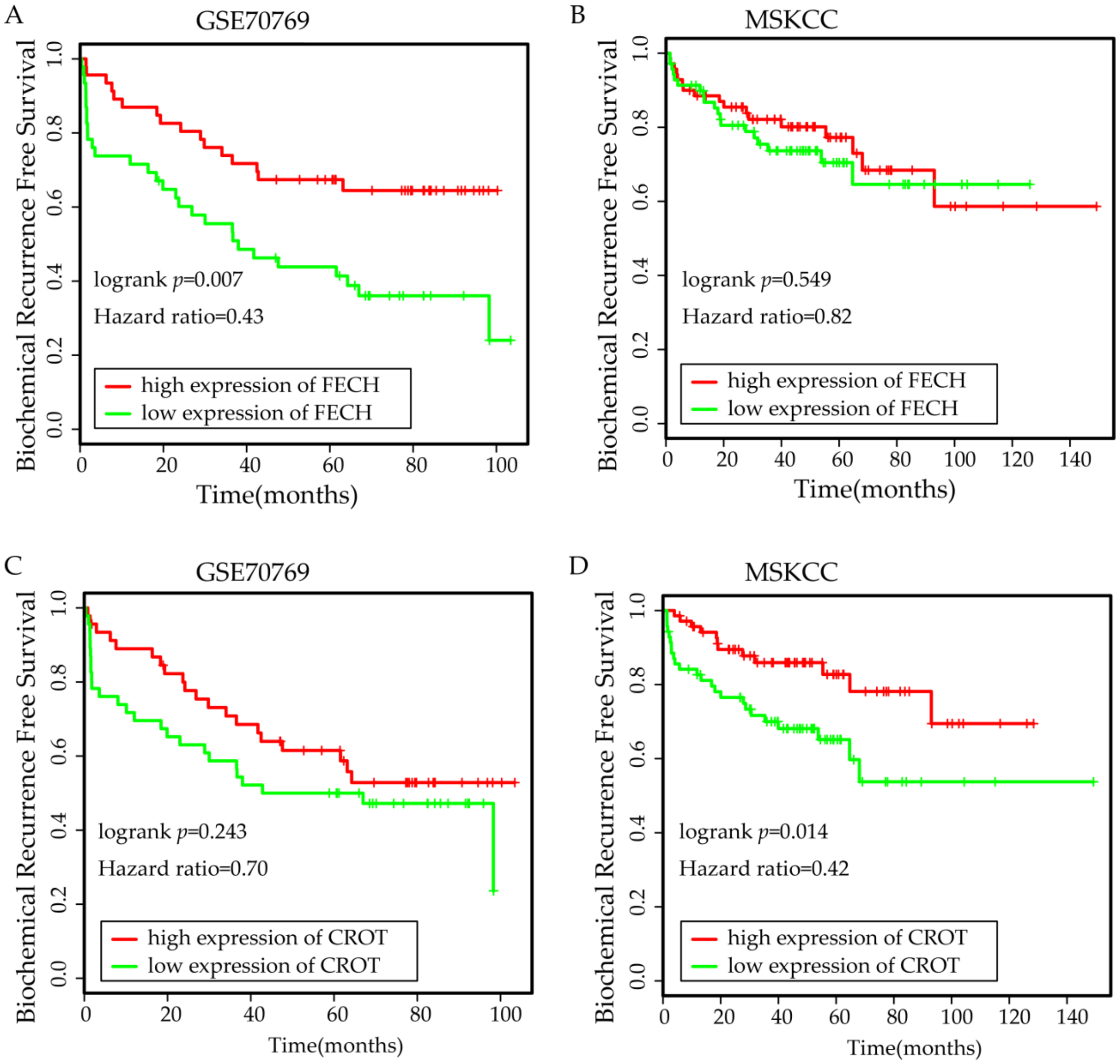

Figure S2. Survival analysis for FECH and CROT. (**A**) FECH is a potent prognostic marker of prostate cancer based on survival analysis of the dataset GSE70769 but not (**B**) the dataset MSKCC. (**C**) CROT is not a potent prognostic marker of prostate cancer based on survival analysis of the dataset GSE70769. (**D**) CROT is a prognostic marker based on survival analysis of the dataset MSKCC.
